# Relationship between the shape of intracranial pressure pulse waveform and computed tomography characteristics in patients after traumatic brain injury

**DOI:** 10.1186/s13054-023-04731-z

**Published:** 2023-11-17

**Authors:** Agnieszka Kazimierska, Agnieszka Uryga, Cyprian Mataczyński, Marek Czosnyka, Erhard W. Lang, Magdalena Kasprowicz, CENTER-TBI high-resolution sub-study participants and investigators, Audny Anke, Ronny Beer, Bo-Michael Bellander, Erta Beqiri, Andras Buki, Manuel Cabeleira, Marco Carbonara, Arturo Chieregato, Giuseppe Citerio, Hans Clusmann, Endre Czeiter, Bart Depreitere, Ari Ercole, Shirin Frisvold, Raimund Helbok, Stefan Jankowski, Daniel Kondziella, Lars-Owe Koskinen, Ana Kowark, David K. Menon, Geert Meyfroidt, Kirsten Moeller, David Nelson, Anna Piippo-Karjalainen, Andreea Radoi, Arminas Ragauskas, Rahul Raj, Jonathan Rhodes, Saulius Rocka, Rolf Rossaint, Juan Sahuquillo, Oliver Sakowitz, Peter Smielewski, Nino Stocchetti, Nina Sundstrom, Riikka Takala, Tomas Tamosuitis, Olli Tenovuo, Andreas Unterberg, Peter Vajkoczy, Alessia Vargiolu, Rimantas Vilcinis, Stefan Wolf, Alexander Younsi, Frederick A. Zeiler

**Affiliations:** 1 Department of Biomedical Engineering, Faculty of Fundamental Problems of Technology, Wroclaw University of Science and Technology, 27 Wybrzeze Wyspianskiego Street, 50-370 Wroclaw, Poland; 2 Department of Computer Engineering, Faculty of Electronics, Wroclaw University of Science and Technology, Wroclaw, Poland; 3 Brain Physics Laboratory, Division of Neurosurgery, Department of Clinical Neurosciences, University of Cambridge, Cambridge, UK; 4 Institute of Electronic Systems, Faculty of Electronics and Information Technology, Warsaw University of Technology, Warsaw, Poland; 5 Neurosurgical Associates, Red Cross Hospital, Kassel, Germany; 6 Department of Neurosurgery, Faculty of Medicine, Georg-August-Universität, Göttingen, Germany

**Keywords:** Intracranial pressure, Pulse waveform, Morphological analysis, Traumatic brain injury, Computed tomography, Neuromonitoring

## Abstract

**Background:**

Midline shift and mass lesions may occur with traumatic brain injury (TBI) and are associated with higher mortality and morbidity. The shape of intracranial pressure (ICP) pulse waveform reflects the state of cerebrospinal pressure–volume compensation which may be disturbed by brain injury. We aimed to investigate the link between ICP pulse shape and pathological computed tomography (CT) features.

**Methods:**

ICP recordings and CT scans from 130 TBI patients from the CENTER-TBI high-resolution sub-study were analyzed retrospectively. Midline shift, lesion volume, Marshall and Rotterdam scores were assessed in the first CT scan after admission and compared with indices derived from the first 24 h of ICP recording: mean ICP, pulse amplitude of ICP (AmpICP) and pulse shape index (PSI). A neural network model was applied to automatically group ICP pulses into four classes ranging from 1 (normal) to 4 (pathological), with PSI calculated as the weighted sum of class numbers. The relationship between each metric and CT measures was assessed using Mann–Whitney *U* test (groups with midline shift > 5 mm or lesions > 25 cm^3^ present/absent) and the Spearman correlation coefficient. Performance of ICP-derived metrics in identifying patients with pathological CT findings was assessed using the area under the receiver operating characteristic curve (AUC).

**Results:**

PSI was significantly higher in patients with mass lesions (with lesions: 2.4 [1.9–3.1] vs. 1.8 [1.1–2.3] in those without; *p* << 0.001) and those with midline shift (2.5 [1.9–3.4] vs. 1.8 [1.2–2.4]; *p* < 0.001), whereas mean ICP and AmpICP were comparable. PSI was significantly correlated with the extent of midline shift, total lesion volume and the Marshall and Rotterdam scores. PSI showed AUCs > 0.7 in classification of patients as presenting pathological CT features compared to AUCs ≤ 0.6 for mean ICP and AmpICP.

**Conclusions:**

ICP pulse shape reflects the reduction in cerebrospinal compensatory reserve related to space-occupying lesions despite comparable mean ICP and AmpICP levels. Future validation of PSI is necessary to explore its association with volume imbalance in the intracranial space and a potential complementary role to the existing monitoring strategies.

## Background

Traumatic brain injury (TBI) is a complex clinical entity that encompasses both the primary insult and a large number of secondary complications [1]. Midline shift and mass lesions are two factors whose presence strongly influences outcome after TBI [2, 3]. Midline shift is the displacement of brain tissue across the center line of the brain which indicates a significant mass effect in the brain structures. The term ‘mass lesions’ applies to a wide range of localized injuries with large volume that may develop after TBI, including hematomas, contusions and hemorrhages. Those two factors are commonly assessed in computed tomography (CT) examinations of TBI patients, for instance in the Marshall classification [4] and the Rotterdam scale [5], as they serve as indicators for surgical evacuation of space-occupying lesions and/or decompressive craniectomy (DC) [6–9].

As mass lesions and midline shift interrupt the volume balance within the intracranial space, they may be associated with elevation of intracranial pressure (ICP) [10]. Monitoring of mean ICP and cerebral perfusion pressure (CPP) and maintaining them within recommended safe ranges is often employed in the intensive care unit (ICU) as the management strategy for TBI patients [11]. However, the efficacy of this approach in preventing the deterioration of the patient’s condition remains controversial [12–16], as mean ICP alone is not sufficient for comprehensive assessment of the cerebrospinal pressure–volume compensation [12] and its monitoring is probably not able to improve outcome [14]. Cerebrospinal compliance is a metric that describes the ability of the cerebrospinal system to buffer changes in volume without disproportionate changes in pressure [17]. Continuous monitoring of compliance could therefore aid in identifying patients at risk of life-threatening intracranial hypertension before such state occurs. However, direct methods of compliance estimation require direct manipulation of intracranial volume and only allow for intermittent measurement and therefore are poorly suited to the management of TBI patients in the ICU.

Most of the techniques developed to assess compliance indirectly are based on analysis of the ICP pulse waveform, i.e., short-term oscillations in the ICP signal that are related to the cardiac cycle [18, 19]. Continuous assessment of cerebrospinal compensatory reserve has been previously postulated using the RAP index [20, 21]. This method takes into account changes in the fundamental frequency pulse amplitude and correlates them with slow fluctuations in ICP. Therefore, it neglects the morphology of the ICP pulse waveform. On the other hand, it has been suggested that progressive change in the pulse shape from a three-phasic, saw-tooth pattern to a rounded or triangular wave with only one defined maximum corresponds to decreasing compliance [22]. Recently, we proposed a method of analyzing the compliance-related changes in ICP pulse morphology that uses a deep neural network model to classify characteristic waveform shapes on a four-category scale ranging from normal to pathological [23]. Our preliminary studies in a small cohort of TBI patients showed that patients with poor outcome exhibited significantly fewer normal waveforms and more pathologically altered waveforms than those with good outcome. This can be seen even at relatively low mean ICP levels (< 20 mm Hg) and in the absence of differences in ICP pulse amplitude or the RAP index [23, 24]. Based on the classification results, we introduced a summary measure called the pulse shape index (PSI). In the large, multi-center dataset of the CENTER-TBI project we showed that PSI was significantly higher in patients with poor outcome and was a significant predictor of mortality [25]. The same study also assessed three other pulse shape-related metrics that are believed to reflect cerebrospinal compliance and showed that pulse amplitude of ICP and the high-frequency centroid may be useful in outcome prediction, while the higher harmonics centroid may help in prediction of intracranial hypertension. In the present study we aimed to investigate whether the shape of the ICP pulse waveform reflects volume imbalance in the intracranial space associated with the presence of midline shift and mass lesions as well as its relationship with the Marshall and Rotterdam scores.

## Methods

### Data acquisition

This study was conducted as a retrospective analysis of data collected in the high-resolution sub-study of the CENTER-TBI project (https://www.center-tbi.eu/; ClinicalTrials.gov identifier NCT02210221), with approval from the CENTER-TBI committee (Approval No. 359). ICP was measured using intraparenchymal strain gauge probes (Codman ICP MicroSensor, Codman & Shurtleff Inc., Raynham, MA, USA) or parenchymal fiber optic pressure sensors (Camino ICP Monitor, Integra Life Sciences, Plainsboro, NJ, USA). The signal was recorded with sampling frequency of 100 Hz or higher using ICM + software (Cambridge Enterprise Ltd., Cambridge, UK) and/or Moberg CNS Monitor (Moberg Research Inc., Ambler, PA, USA). Data for the CENTER-TBI study were collected through Quesgen e-CRF (Quesgen Systems Inc., USA), hosted on the INCF platform and extracted via the INCF Neurobot tool (INCF, Sweden). Version CENTER Core 3.0 of the CENTER-TBI dataset was used in this study.

The CENTER-TBI study (European Commission grant 602150) was conducted in accordance with all relevant laws of the European Union if directly applicable or of direct effect and all relevant laws of the country where the recruiting sites were located, including but not limited to, the relevant privacy and data protection laws and regulations (the “Privacy Law”), the relevant laws and regulations on the use of human materials, and all relevant guidance relating to clinical studies from time to time in force including, but not limited to, the ICH Harmonised Tripartite Guideline for Good Clinical Practice (CPMP/ICH/135/95) (“ICH GCP”) and the World Medical Association Declaration of Helsinki entitled “Ethical Principles for Medical Research Involving Human Subjects.” Informed consent by the patients and/or the legal representative/next of kin was obtained, accordingly to the local legislations, for all patients recruited in the Core Dataset of CENTER-TBI and documented in the e-CRF. Ethical approval was obtained for each recruiting site from the appropriate local ethics committee, and the full list of approvals is available on the website: https://www.center-tbi.eu/project/ethical-approval.

### Study population

**Fig. 1:**
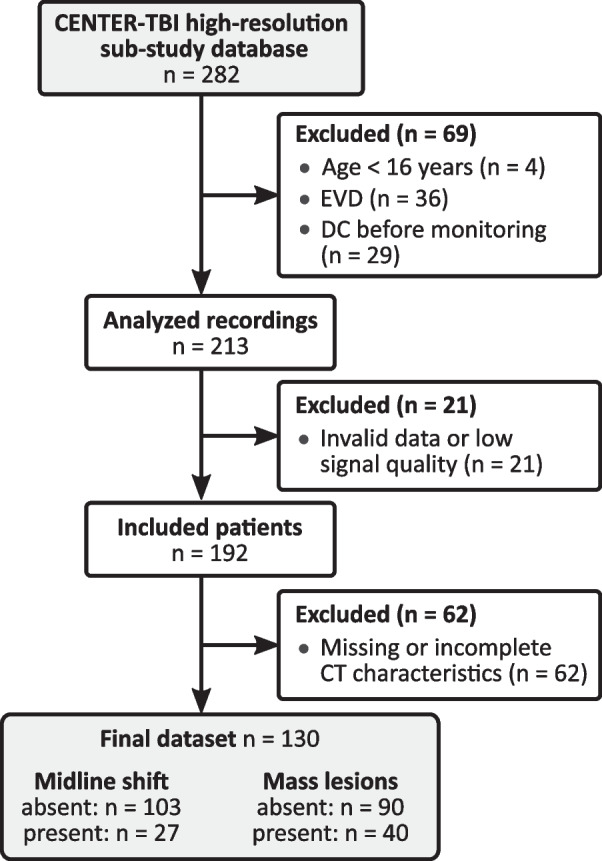
Selection criteria for the final patient dataset included in the study. Patients with external ventricular drains (EVDs) were excluded due to unavailability of the intracranial pressure pulse waveform during drainage. Patients who underwent decompressive craniectomy (DC) before the start of monitoring were excluded due to disturbance of the intracranial pressure–volume conditions. We also excluded patients with missing or incomplete computed tomography (CT) characteristics (no admission CT scan available and those with uninterpretable CT examinations). *n*—number of patients

The initial dataset consisted of 282 patients. The selection criteria are presented in Fig. 1. Patients in whom ICP was measured via external ventricular drains (EVDs) were excluded because the ICP pulse waveform was not available for assessment during cerebrospinal fluid (CSF) drainage periods. Patients in whom DC was performed before the start of ICP monitoring were excluded due to the alteration in the intracranial pressure–volume relationship caused by removal of a fragment of the skull boundary. Detailed summary of the study population is presented in the Results section.

### Computed tomography examinations

For each patient the CT scan performed directly prior to the start of monitoring (‘early CT’) was selected as the basis for assessment. Both the shift of the central line of the brain (expressed in mm) and the total volume of all lesions (expressed in cm^3^) were measured in the scan. Midline shift was considered present if a shift exceeding 5 mm was observed. Mass lesions were considered present if the total volume exceeded 25 cm^3^; this could indicate either one large lesion or multiple coexisting lesions that added up to that volume. Moreover, the patients were assessed using the Marshall [4] and Rotterdam [5] scores. Those with missing CT information were excluded from further analyses (see Fig. 1). While mass lesions and midline shift are not mutually exclusive conditions and may occur in the same patient (here, 22 patients presented both pathological features), they were analyzed separately to investigate whether either is more strongly linked with alterations in ICP pulse shape. Detailed summary of the CT characteristics is presented in the Results section.

### Intracranial pressure monitoring

In each patient the first 24 h of recording was used to calculate metrics describing the ICP signal: mean value, peak-to-peak pulse amplitude of ICP (AmpICP), and PSI (see “Assessment of intracranial pressure pulse waveform morphology” section). This initial period was selected to analyze data most closely corresponding to the time the CT scans were taken. In most patients monitoring commenced on day 1 (21%) or day 2 (65%) postinjury and the time elapsed between the CT scan and the start of ICP monitoring did not exceed 48 h in 98% of the patients (in 67% monitoring began within 24 h of the scan).

### Assessment of intracranial pressure pulse waveform morphology

**Fig. 2:**
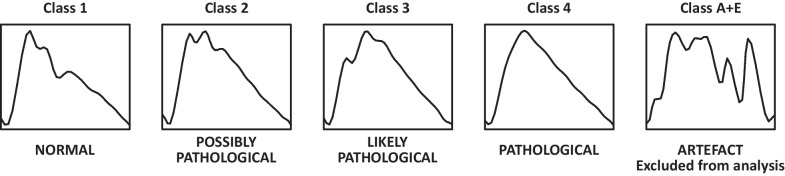
Illustrative examples of intracranial pressure pulse waveforms with different morphologies. Each figure shows an example of waveform assigned to one of the five classes identified by the neural network model (four valid pulse types ranging from 1 to 4 and the fifth type representing artifacts and errors in pulse detection)

The association between the shape of ICP pulse waveform and cerebrospinal compliance has been suggested in earlier studies [22, 26, 27] and recently validated in hydrocephalus patients undergoing controlled changes in mean ICP [28]. It has been demonstrated that as cerebrospinal compensatory reserve is reduced, the second peak of the ICP waveform becomes more prominent, eventually overtaking the first and leading to a rounded or triangular pulse. Based on the results of these studies, we developed a deep neural network model to classify characteristic shapes of ICP pulses observed in TBI patients [23]. The model identifies four types of pulse waveforms that have been shown to correspond to a progressive reduction in compliance and represent changes in the relative height and visibility of characteristic peaks P1, P2, and P3 [22] (see Fig. 2). These are: normal pulse (class 1, with dominant peak P1), potentially pathological (class 2, with increased prominence of peak P2 but P1 higher than P3), likely pathological (class 3, with increased prominence of both P2 and P3), and pathological (class 4, rounded or triangular waveforms with only one visible maximum). Additionally, distorted waveforms or errors in pulse detection are simultaneously marked as artifacts in order to exclude invalid parts of the recording from further analyses.

The algorithm was trained in over 20,000 manually classified waveforms extracted from recordings of TBI patients, achieving classification accuracy of 93%, and validated in an independent external dataset of patients with aneurysmal subarachnoid hemorrhage (classification accuracy 82%). Details of the development and validation process are presented in our previous work [23].

**Fig. 3:**
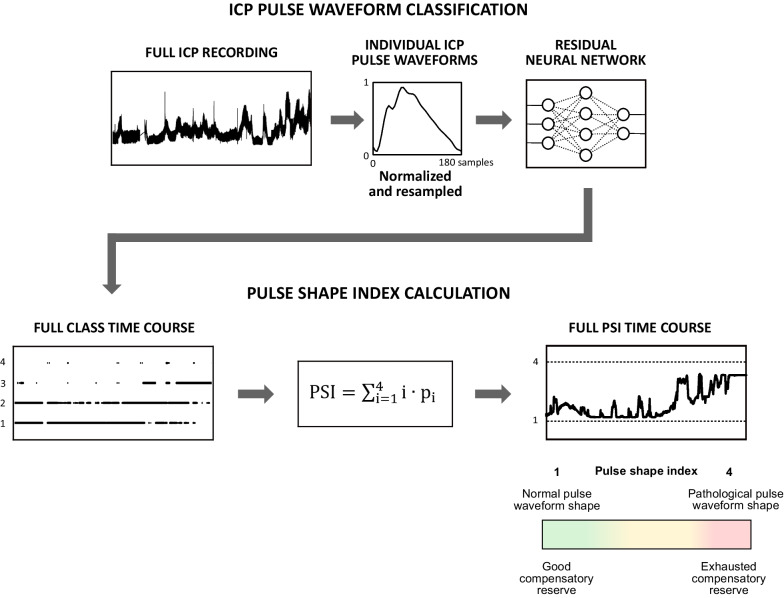
Schematic representation of the methodology to obtain the pulse shape index (PSI). Classification results for individual waveforms are obtained from full intracranial pressure (ICP) recording using a deep neural network model. PSI represents the mean class number in a 5-min moving window (shifted every 10 s) and is expressed as the weighted sum of class numbers *i* with weights corresponding to the fraction of pulses assigned to given class *p*_*i*_*,* where *i* = {1, 2, 3, 4}, due to the discrete (rather than continuous) scale of class numbers

Here, the classification results produced by the model were used to derive a summary metric called PSI, calculated in moving 5-min windows (window shift: 10 s) as the weighted sum of class numbers *i* and the fraction of pulses assigned to given class *p*_*i*_ (excluding artifacts; see Fig. 3). If considered at single-pulse level, the classification result will only change if the shape moves from one class to another, and monitoring of alterations in pulse morphology over time requires simultaneous tracking of four separate values (e.g., percentage of pulses assigned to given class), which is not ideal for the application in long-term monitoring. In turn, PSI reflects the mean class number in a given period and thus allows for the gradual changes in pulse shape to be captured by a single index expressed on a continuous scale from 1 (only normal waveforms of class 1) to 4 (only pathologically altered waveforms of class 4). For instance, if a patient initially exhibits only normal triphasic ICP waveforms, but the prominence of peak P2 starts to increase to the point where it exceeds P1 in half of the pulses, this would register as a change in PSI of approximately 0.5 (from 1.0 to 1.5). Furthermore, the use of an automatic machine learning-based classification algorithm at single-pulse level allows for even small changes in the occurrence of different pulse classes to be directly detected as an alteration in PSI.

### Statistical analysis

Statistical analysis was performed using Statistica software (v13, Tibco, Palo Alto, CA, USA) and custom-written programs in Python 3.8. Normality of data distributions was tested using the Shapiro–Wilk test with significance level of 0.05. Upon rejection of the normality hypothesis for most of considered variables, nonparametric measures were chosen in subsequent analyses. The relationship between ICP-derived indices and quantitative CT characteristics (the extent of midline shift and total lesion volume) as well as Marshall and Rotterdam scores was assessed using the Spearman correlation coefficient. The Mann–Whitney *U* test was used to assess the differences in ICP-derived metrics between patients with and without midline shift and mass lesions. Performance of ICP-derived metrics as binary classifiers for the presence or absence of pathological changes in CT scans was assessed using area (AUC) under the receiver operating characteristic (ROC) curve, including estimation of the classification threshold. The significance level of 0.05 was used in all analyses. All results are presented as median [first–third quartile] unless stated otherwise.

## Results

### Patient characteristics

Out of the full dataset of 282 patients, 130 were included in the analysis. 77% of the patients were male and the patients’ age ranged from 16 to 82 years. With median admission Glasgow Coma Score (GCS) of 6 [3–10], the group’s condition was classified as moderate to severe, with majority of patients assessed as severely injured (scores 3–9: 65%). Summary clinical characteristics of the patient cohort are presented in Table 1, while detailed CT characteristics are reported in Table 2.

**Table 1:** Summary clinical characteristics of the patient cohort

Parameter	Value *total n* = *130*
Age [years] *median [Q1–Q3]*	46 [28–59]
Sex *n*	Female: 30, male: 100
GCS score at admission *median [Q1–Q3]*	6 [3–10], NA: 8
Pupil reactivity at admission *n*	Bilaterally reactive: 62, unilaterally reactive: 5, bilaterally nonreactive: 11, NA: 52
ICU mortality *n*	Survived: 121, deceased: 9
Mortality after 6 months (GOSE score 1: deceased, 2–8: survived) *n*	Survived: 99, deceased: 18, NA: 13

Data are presented as number of occurrences (*n*) or as median [first–third quartile]

*Q1* first quartile, *Q3* third quartile, *NA* data not available, *GCS* Glasgow Coma Scale, *ICU* intensive care unit, *GOSE* Glasgow Outcome Scale Extended

**Table 2:** Computed tomography (CT) characteristics of the patient cohort

CT characteristic	Value *total n* = *130*
Midline shift *n (%)*	Absent: 103 (79%), present: 27 (21%)
Mass lesions *n (%)*	Absent: 90 (69%), present: 40 (31%)
Both midline shift and mass lesions present *n (%)*	22 (17%)
Marshall classification:	
Category I *n (%)*	3 (2.5%)
Category II *n (%)*	69 (53%)
Category III *n (%)*	17 (13%)
Category IV *n (%)*	3 (2.5%)
Category V *n (%)*	0 (0%)
Category VI *n (%)*	38 (29%)
Rotterdam score *median [Q1–Q3]*	3 [3–4]
Score 1 *n (%)*	1 (1%)
Score 2 *n (%)*	18 (14%)
Score 3 *n (%)*	64 (49%)
Score 4 *n (%)*	21 (16%)
Score 5 *n (%)*	22 (17%)
Score 6 *n (%)*	4 (3%)

Data are presented as number of occurrences (*n*) with percentage of the full group or as median [first–third quartile]

*Q1* first quartile, *Q3* third quartile

### Relationship between ICP-derived metrics and binary CT characteristics

Table 3 presents the comparison of mean ICP, AmpICP, and PSI values between patients with and without midline shift and with and without mass lesions. Only PSI statistically differentiated patients with midline shift or mass lesions from those without; mean ICP was slightly but not significantly higher in patients with either of the pathological changes observed in CT scans, while AmpICP was comparable in the presence of midline shift and slightly elevated in patients with mass lesions.

**Table 3:** Comparison of intracranial pressure (ICP)-derived metrics between patients with and without midline shift/mass lesions

Parameter	Midline shift			Mass lesions		
	Absent *n* = 103	Present *n* = 27	*p* value	Absent *n* = 90	Present *n* = 40	*p* value
Mean ICP [mm Hg]	12.2 [9.4–15.8]	13.5 [11.4–17.1]	n.s	12.2 [9.1–15.6]	13.3 [10.7–16.8]	n.s
AmpICP [mm Hg]	8.6 [6.7–10.5]	8.9 [6.8–10.8]	n.s	8.4 [6.6–10.4]	9.4 [7.2–11.5]	n.s
PSI [a.u.]	1.8 [1.2–2.4]	2.5 [1.9–3.4]	< 0.001	1.8 [1.1–2.3]	2.4 [1.9–3.1]	<< 0.001

Data are presented as median [first–third quartile] with Mann–Whitney *U* test *p* value

*AmpICP* peak-to-peak pulse amplitude of ICP, *PSI* pulse shape index, *a.u.* arbitrary units, *n* number of patients, *n.s.* result not statistically significant

### ICP-derived metrics as indicators of mass lesions and midline shift

**Fig. 4:**
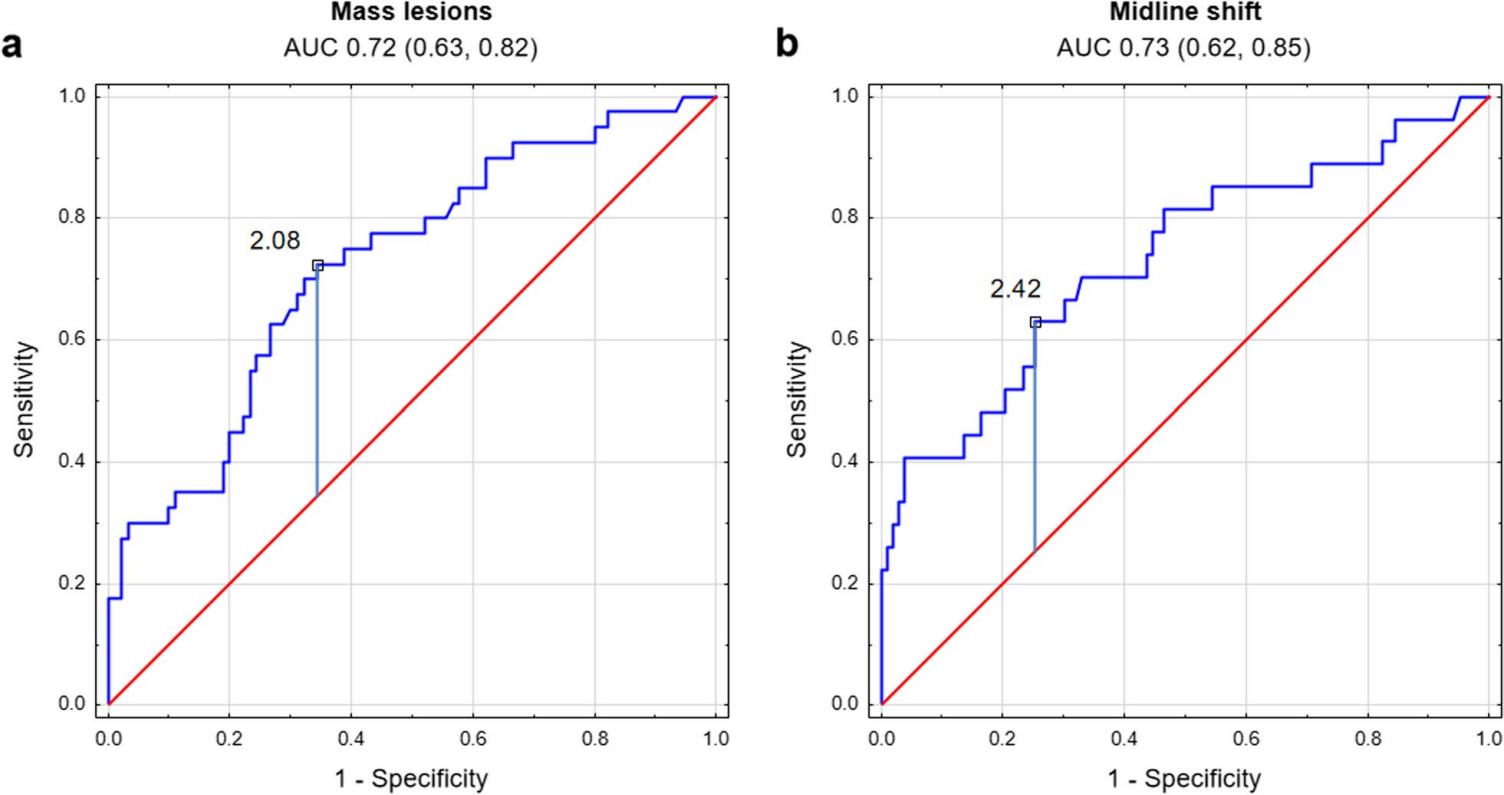
ROC curves for binary classification of patients based on pulse shape index (PSI) value. Subplots show the results of patient classification as presenting or not presenting **a** mass lesions or **b** midline shift. Area under the curve (AUC) is presented above the plot with 95% confidence interval. Vertical blue line denotes the value estimated as best-performing PSI threshold

Mean PSI level used as a binary classifier to determine whether a patient exhibits pathological changes in the brain CT scans showed AUC (presented with 95% confidence intervals) of 0.72 (0.63, 0.82) for mass lesions and 0.73 (0.62, 0.85) for midline shift. Both ROC curves are shown in Fig. 4. PSI threshold for patient classification as presenting mass lesions was estimated at 2.08 which yielded accuracy of 0.68, sensitivity of 0.73, and specificity of 0.66. For midline shift, the estimated cutoff threshold was 2.42, yielding accuracy of 0.72, sensitivity of 0.63, and specificity of 0.75. The AUC for detection of mass lesions present was significantly higher for PSI than those for mean ICP and AmpICP (0.60 (0.49, 0.70) and 0.58 (0.47, 0.69), respectively) and higher than AmpICP (0.51 (0.38, 0.64)) for detection of midline shift. While the AUC for detection of midline shift was not statistically different for PSI and mean ICP (0.60 (0.49, 0.72)), the overall performance was less balanced, with higher sensitivity (0.78) but lower accuracy (0.52) and specificity (0.46).

### Relationship between ICP-derived metrics and quantitative CT metrics

**Fig. 5:**
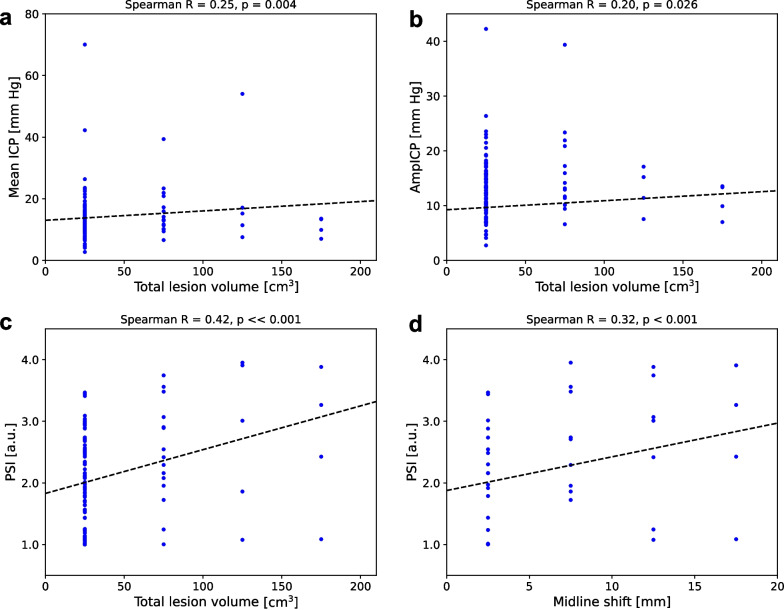
Significant relationships between intracranial pressure (ICP)-derived metrics and quantitative measures of computed tomography features. **a** Mean ICP versus total lesion volume. **b** Peak-to-peak pulse amplitude of ICP (AmpICP) versus total lesion volume. **c** Pulse shape index (PSI) versus total lesion volume. d) PSI versus the extent of midline shift. Individual observations (dots) are grouped over total lesion volume (bin width 50 cm^3^) or the extent of midline shift (bin width 5 mm). Dashed line shows the estimated linear regression line. Values above the plots indicate the Spearman correlation coefficient and its *p* value. a.u.—arbitrary units

Figure 5 presents the relationship between ICP-derived metrics and quantitative measures of midline shift and total lesion volume. The correlation between the extent of midline shift and total lesion volume was strongest for PSI, although in general the correlation was moderate (Spearman correlation coefficient of 0.32, *p* < 0.001 for midline shift and 0.42, *p* << 0.001 for lesion volume). Mean ICP and AmpICP were correlated with total lesion volume (correlation coefficients of 0.25, *p* = 0.004 and 0.20, *p* = 0.026, respectively), but not with the extent of midline shift.

### Relationship between ICP-derived metrics and the Marshall and Rotterdam scores

**Fig. 6:**
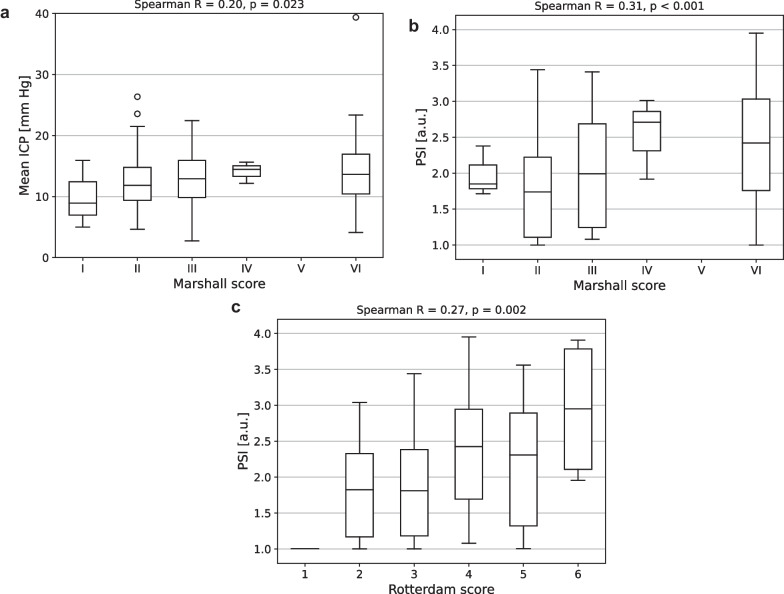
Significant relationships between intracranial pressure (ICP)-derived metrics and the summary scores of computed tomography examinations. **a** Mean ICP versus Marshall score. **b** Pulse shape index (PSI) versus Marshall score. **c** PSI versus Rotterdam score. Data are shown as median (central lines) and interquartile range (boxes), with whiskers extending to minimum and maximum values not including outliers (shown as circles). Extreme values are not shown for readability. Values above the plots indicate the Spearman correlation coefficient and its *p* value. Please note that no patients were assigned to Marshall category V and only one patient had Rotterdam score of 1. a.u.—arbitrary units

Both higher Marshall score and higher Rotterdam score were associated with elevated PSI values (Spearman correlation coefficients of 0.31, *p* < 0.001 and 0.27, *p* = 0.002, respectively). Mean ICP was weakly correlated with the Marshall score (correlation coefficient of 0.20, *p* = 0.023) but not the Rotterdam score, whereas AmpICP showed no significant correlation with either metric. The significant relationships between ICP-derived indices and the Marshall and Rotterdam scores are presented in Fig. 6. While PSI differed visibly between Rotterdam scores 1 and 6, the values for scores 2 and 3 as well as 4 and 5 were comparable, and a similar pattern was observed for Marshall classification categories I–III and IV and VI, which likely contributed to the relatively low correlation coefficients; no patients were assessed with Marshall score V. AmpICP did not follow any specific trends, as expected from the lack of significant correlation.

### Discussion

In this study we investigated the relationship between the shape of ICP pulse waveform expressed using PSI and the presence of pathological features in CT scans of TBI patients as well as the Marshall and Rotterdam scores used in summary assessment of the patients’ CT examinations. The results show that both mass lesions and midline shift are associated with significant elevation in PSI and this relationship is stronger than corresponding changes in mean ICP or pulse amplitude of ICP. This is in line with our previous studies [23, 25] which suggested that ICP pulse shape in TBI is not dependent solely on mean ICP, but pathological changes in the waveform can be observed in the absence of intracranial hypertension.

The changing configuration and visibility of characteristic peaks in the ICP pulse contour which is the basis for pulse classification used to calculate PSI has long been linked to reductions in cerebrospinal compliance [22, 26, 27]. Mass lesions and midline shift may be considered as a substantial disruption of the intracranial volume equilibrium which under normal conditions is governed by the relationship between brain tissue, CSF, and cerebral blood [29]. In general, taking into account the nonlinear relationship between intracranial volume and pressure expressed by the pressure–volume (P–V) curve [30, 31], a sufficiently large increase in intracranial volume is expected to shift the patient toward the steeply rising region of the P–V curve and result in an increase in mean ICP as well as AmpICP [32]. Elevated mean ICP is therefore invariably a sign of reduced cerebrospinal compliance, but the disturbance in the pressure–volume relationships is thought to occur even before an increase in mean ICP can be observed. Most patients included in this study exhibited ICP levels below 22 mm Hg (i.e., the current guidelines-supported threshold for intracranial hypertension [11]) in the analyzed period regardless of the result of their CT scans, and there were no statistically significant differences in mean ICP between patients with midline shift or mass lesions and those without, in contrast to detected changes in ICP pulse morphology.

Continuous monitoring of cerebrospinal compliance is considered potentially beneficial in the management of TBI as it could allow for early identification of patients with disturbed intracranial volume equilibrium who are at risk of associated adverse effects. However, direct estimates of compliance, such as the volume–pressure response (VPR) [33] or the pressure–volume index (PVI) [17], require invasive manipulation of intracranial volume in order to assess the pressure change for a given volume increment. This could produce uncontrolled changes in mean ICP and endanger the life of a neurocritical care patient already at risk of intracranial hypertension. Indirect measures of compliance proposed over the years [20, 32, 34, 35] are mostly based on analysis of the ICP pulse waveform which is considered the pressure response to short-term volume increments related to cerebral blood inflow and subsequent outflow during the cardiac cycle [18]. While they do not express compliance in absolute units of milliliters per millimeter mercury due to the unknown cerebral fraction of cardiac stroke volume, they could allow for continuous monitoring not permitted by the direct measures. However, to date there is no agreement whether different indirect estimates reflect cerebrospinal pressure–volume compensation in the same way. Recently, we showed that while AmpICP, PSI, and a spectral index called the high-frequency centroid are correlated with mortality after TBI, a different spectral measure, the higher harmonics centroid, may be more useful in prediction of intracranial hypertension [25], highlighting the differences between these indices. In this study, we observed that changes in ICP pulse morphology reflected by PSI may be more sensitive to disruption of intracranial volume balance than AmpICP, as AmpICP did not differ significantly between patients with midline shift or mass lesions and those without and was not correlated with either the Marshall or Rotterdam score. This suggests that the information on volume imbalance in the intracranial space is encoded in the shape of the ICP pulse waveform rather than just its pulse amplitude.

One previous study that assessed cerebrospinal compliance using VPR calculated from repeated bolus injections showed that VPR is correlated with the degree of midline shift in TBI patients [36]. As changes in cerebrospinal compliance are expected to predate changes in mean ICP, the increase in PSI associated with the presence of mass lesions and midline shift, i.e., disturbed intracranial volume equilibrium, that we observed in this study supports the view that ICP pulse morphology at least partially reflects the state of cerebrospinal pressure–volume compensation. Results of this study also show that elevated PSI value performs relatively well in binary classification of TBI patients as presenting mass lesions or midline shift compared to standard ICP-derived metrics, i.e., mean ICP and its pulse amplitude, and is more strongly linked to the Marshall and Rotterdam scores. The slightly different pattern of changes in PSI over the two scales can likely be attributed to the differences in their definition. As the Marshall score is based on binary descriptors of midline shift and mass lesions but also the visibility of basal cisterns and surgical evacuation of lesions, its value represents different types of pathological CT findings rather than their gradually increasing extent reflected in the more quantitative Rotterdam score. Correspondingly, the increase in PSI from Rotterdam score 1 to 6 appears unidirectional, possibly reflecting the progressive disruption of the intracranial volume balance.

CT imaging is a powerful diagnostic tool widely used in the management of TBI. The Traumatic Coma Data Bank CT classification, which defines, among others, the thresholds for mass lesions and midline shift as used in this study, has been shown to correlate with outcome after TBI [4, 37], as have various individual CT characteristics and combinations thereof [5, 38–42]. However, at present neither CT nor related imaging methods, e.g., magnetic resonance imaging, can be used in continuous monitoring at the bedside. This presents an important drawback as the condition of TBI patients may deteriorate rapidly and successful therapeutic intervention depends on early detection of life-threatening changes such as developing space-occupying lesions. High-resolution ICP monitoring is commonly employed in modern ICUs, making it a readily available source of additional information on the state of the cerebrospinal space. Still, so far analysis of the shape of the ICP pulse waveform with automated tools has not achieved widespread use in the clinical setting. PSI as a summary measure of ICP pulse morphology has shown promise in identifying patients with disturbed cerebrospinal compensatory reserve. This approach could act as a complementary continuous assessment technique to identify patients suspected of developing pathological alterations in the brain which could then be confirmed by imaging methods as necessary. As shown by this study, elevated PSI value, i.e., pathologically altered shape of the ICP pulse waveform, is associated with larger extent of midline shift, larger lesion volume, and higher Marshall and Rotterdam scores.

#### Limitations

This study was performed as a retrospective analysis of an existing multi-center dataset. Due to the constraints of the management strategy for TBI employed in the original project, the early CT scans were taken some time before the start of ICP monitoring. Although we selected the analysis period, i.e., the first 24 h of ICP measurement, to most closely correspond to the time of the CT scan, we acknowledge that the values estimated from ICP recording are from a different timepoint. Consequently, this study could not assess how far in advance a change in PSI can be detected with regard to development of mass lesions or midline shift. Further investigation is required to determine if a specific pattern of changes in the ICP pulse waveform shape can be observed before pathological changes are confirmed by brain imaging. Moreover, as TBI is a complex condition with insults not limited to mass lesions and midline shift, other factors that could contribute to volume imbalance in the cerebrospinal system and register as changes in ICP pulse morphology should be included in the future, e.g., post-traumatic hydrocephalus or alterations in cerebral blood flow. Taking into account the preliminary nature of this study, we chose to restrict the analysis to mass lesions and midline shift as those two features are very significant for the management of TBI patients.

Secondly, the proposed approach to ICP pulse waveform shape assessment requires a high-quality ICP signal recording. Based on our observations as well as a previous study by Holm and Eide [43], 50 Hz is the minimum viable sampling frequency to obtain a sufficiently high resolution of the ICP pulse waveform to reliably evaluate the pulse shape. Moreover, in this study we analyzed intraparenchymal ICP measurements from the Codman and Camino sensors as those two types of devices were used to collect the CENTER-TBI dataset; the information on precise sensor type in individual cases was not available and we could not assess the possible differences between the two. However, previous studies reported that the Codman and Camino sensors produce comparable single-pulse amplitude and latency (time to systolic maximum) [44] and differences in insertion site between two sensors mostly influence mean ICP rather than pulse shape-related metrics [45], which suggests that the pulse waveform should be comparable. Hence, provided that a sufficient quality of the recording can be achieved, the PSI approach should be applicable to data obtained using different intraparenchymal sensors without modification. Its usefulness in the analysis of ICP recordings collected using EVDs is however limited by the fact that the ICP pulse waveform is not visible (or, if visible, heavily distorted) during drainage periods. Therefore, it is only possible to obtain PSI when the drain is closed which may substantially reduce the amount of usable data and is the reason why we excluded EVD recordings from analysis in this study. Additionally, the agreement between intraventricular and intraparenchymal pulse waveforms remains to be investigated, as existing evidence mostly reported agreement with regard to mean ICP [46–49], with only a single patient study showing comparable single-pulse amplitude and latency [49]. Furthermore, caution should be taken when comparing the results of ICP pulse analysis between different patient positions. While the reduction in mean ICP that accompanies head elevation is well documented, there is relatively little available information on the effect on cerebrospinal compliance and the morphology of ICP pulse waveform. Previous studies suggested that the positional change from supine to upright results in a downward shift of the entire P–V curve due to hydrostatic pressure offset [50, 51], but there is no change in compliance metrics PVI and VPR [50]. However, it remains to be studied to what extent the body and head position influence ICP pulse shape.

With regard to the methodology used to assess ICP pulse waveform shape, it should also be noted that PSI is a summary measure derived from automatic pulse classification using an artificial intelligence algorithm. Our previous study showed that the accuracy of the model reaches 93% in the validation dataset and 82% in an independent testing dataset, confirming good generalization ability [23]. Furthermore, in the training process we observed that incorrect classification primarily concerns adjacent class, with rare cases of normal waveforms categorized as fully pathological or vice versa. However, a small number of classification errors likely could not be avoided in this approach.

### Conclusions

Continuous monitoring of ICP pulse shape using automatic morphological classification based on the use of artificial intelligence algorithms is feasible. Further validation of PSI is necessary to explore its relationship with changes in volume imbalance in the intracranial space and a potential complementary role to the existing monitoring strategies.

## Data Availability

The data that support the findings of this study belong to the CENTER-TBI project (https://www.center-tbi.eu/) but restrictions apply to the availability of these data, which were used under license for the current study (Approval No. 359), and so are not publicly available. Access to the data can be obtained upon approval from the CENTER-TBI project committee. Source codes and weights for the ICP pulse waveform classification model are available in: https://github.com/CMataczynski/ICP_NN.

## Abbreviations

AmpICP: Pulse amplitude of intracranial pressure
AUC: Area under the ROC curve
CSF: Cerebrospinal fluid
CT: Computed tomography
DC: Decompressive craniectomy
EVD: External ventricular drain
GCS: Glasgow Coma Scale
GOSE: Glasow Outcome Scale Extended
ICP: Intracranial pressure
ICU: Intensive care unit
PSI: Pulse shape index
PVI: Pressure–volume index
P–V: Pressure–volume (curve)
ROC: Receiver operating characteristic (curve)
TBI: Traumatic brain injury
VPR: Volume–pressure response
